# CALHM1 deficiency impairs cerebral neuron activity and memory flexibility in mice

**DOI:** 10.1038/srep24250

**Published:** 2016-04-12

**Authors:** Valérie Vingtdeux, Eric H. Chang, Stephen A. Frattini, Haitian Zhao, Pallavi Chandakkar, Leslie Adrien, Joshua J. Strohl, Elizabeth L. Gibson, Makoto Ohmoto, Ichiro Matsumoto, Patricio T. Huerta, Philippe Marambaud

**Affiliations:** 1 Litwin-Zucker Research Center for the Study of Alzheimer’s Disease, The Feinstein Institute for Medical Research, Manhasset, NY 11030, USA; 2 Laboratory of Immune & Neural Networks, The Feinstein Institute for Medical Research, Manhasset, NY 11030, USA; 3 Monell Chemical Senses Center, Philadelphia, PA 19104, USA; 4 Department of Molecular Medicine, Hofstra Northwell School of Medicine, Manhasset, NY 11030, USA

## Abstract

CALHM1 is a cell surface calcium channel expressed in cerebral neurons. CALHM1 function in the brain remains unknown, but recent results showed that neuronal CALHM1 controls intracellular calcium signaling and cell excitability, two mechanisms required for synaptic function. Here, we describe the generation of *Calhm1* knockout (*Calhm1*^−/−^) mice and investigate CALHM1 role in neuronal and cognitive functions. Structural analysis revealed that *Calhm1*^−/−^ brains had normal regional and cellular architecture, and showed no evidence of neuronal or synaptic loss, indicating that CALHM1 deficiency does not affect brain development or brain integrity in adulthood. However, *Calhm1*^−/−^ mice showed a severe impairment in memory flexibility, assessed in the Morris water maze, and a significant disruption of long-term potentiation without alteration of long-term depression, measured in *ex vivo* hippocampal slices. Importantly, in primary neurons and hippocampal slices, CALHM1 activation facilitated the phosphorylation of NMDA and AMPA receptors by protein kinase A. Furthermore, neuronal CALHM1 activation potentiated the effect of glutamate on the expression of c-Fos and C/EBPβ, two immediate-early gene markers of neuronal activity. Thus, CALHM1 controls synaptic activity in cerebral neurons and is required for the flexible processing of memory in mice. These results shed light on CALHM1 physiology in the mammalian brain.

Calcium homeostasis modulator protein 1 (*CALHM1*)^1^ was identified by a tissue-specific gene expression profiling approach^2^, which was designed to screen for genes preferentially expressed in the hippocampus and located on susceptibility loci for Alzheimer’s disease (AD). AD is a neurodegenerative brain disorder characterized by an array of cognitive disturbances, one of the earliest and most prominent being an impairment in the flexible use of episodic memory^3^. *CALHM1* does not appear to be associated with the risk of developing AD^4^, but independent genetic studies have shown that it influences the onset of the disease^1^,^4^. At the mechanistic level and in cell culture systems, CALHM1 controls the accumulation of the amyloid-β (Aβ) peptide^1^,^5^,^6^, a main culprit in AD^7^. Some studies^8^,^9^, but not all^10^, have also reported an association between a CALHM1 variant and Aβ levels in human cerebrospinal fluid. These results support the notion that CALHM1 might be involved in molecular mechanisms relevant to AD pathogenesis and thus warrant further studies aimed at understanding the exact physiology of CALHM1 in the brain.

Despite one report of failed detection of *Calhm1* mRNA in the mouse brain^11^, CALHM1 expression has now been unambiguously demonstrated in human and murine brains, particularly in hippocampal and cortical neurons^1^,^12^,^13^. The physiological function associated with its neuronal expression in the brain, however, remains incompletely understood. CALHM1 is a plasma membrane, voltage-gated calcium-permeable channel that is regulated by extracellular calcium concentration^1^,^13^,^14^. Its expression in different cell systems induces cationic currents and elevates cytoplasmic calcium levels in response to removal of extracellular calcium and its subsequent add-back^1^,^12^ (hereafter termed the calcium add-back condition, CaAB). The response of CALHM1 to CaAB might be highly relevant to synaptic function because transient decreases in extracellular calcium levels are known to occur within the synaptic cleft during neuronal electrical activity^15^. In mouse cortical neurons, CALHM1 responds to CaAB not only by elevating intra-neuronal calcium levels but also by controlling the cell’s conductance and action potential firing^13^. Studies performed in CALHM1-transfected hippocampal HT-22 cells, as well as *Calhm1*^−/−^ primary neurons, have further demonstrated that CALHM1 not only controls calcium influx but also intracellular calcium signal transduction *via* the activation of a kinase signaling cascade involving extracellular signal-regulated kinase-1/2 (ERK1/2)^12^. Thus, CALHM1 plays an important role in cerebral neuronal calcium homeostasis and signaling, as well as in neuronal excitability. Here, we show that CALHM1 deficiency in mice leads to major cognitive and neuronal deficits, manifested by the presence of impairments in memory flexibility and hippocampal long-term potentiation (LTP). These deficits could not be explained by alterations in brain development or brain integrity in adulthood, but could be associated with impairments in neuronal activity signaling.

## Results

### Generation of a constitutive *Calhm1* ^−/−^ mouse line

**Figure 1:**
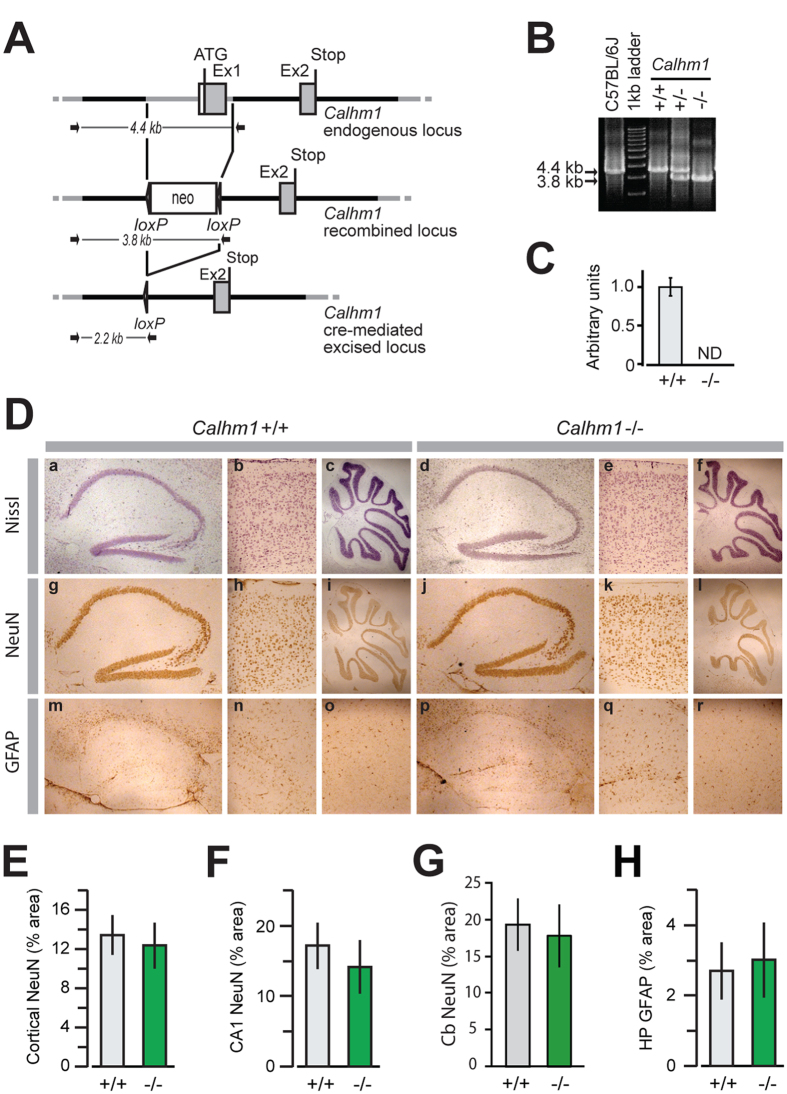
Generation and brain analysis of *Calhm1*^−/−^ mice. (**A**) Schematic representation of the *Calhm1* targeting strategy resulting in exon (Ex) 1 deletion. Grey boxes represent *Calhm1* coding sequences and solid lines the chromosome sequence. The initiation (ATG) and stop (Stop) codons are indicated. Arrows show primers used for genotyping; neo, neomycin cassette. (**B**) PCR genotyping of *Calhm1*^−/−^ mouse lines. PCR was performed on tail genomic DNA of a C57BL/6J mouse control, *Calhm1*^+/+^ (+/+), *Calhm1*^+/−^ (+/−), and *Calhm1*^−/−^ (^−/−^) littermates. Arrows point to 4.4-kb and 3.8-kb PCR products corresponding to amplified regions of *Calhm1* endogenous locus and recombined locus, respectively (as shown schematically in (**A**)). (**C**) Real time PCR analyzing *Calhm1* expression levels in whole brains from *Calhm1*^+/+^ and *Calhm1*^−/−^ mice. *Calhm1* expression was normalized to the reference genes *Hprt1*, *Tbp*, and *Polr2a*; ND, not detected. (**D**) Nissl (panels a–f), NeuN (g–l), and GFAP (m–r) staining of sagittal brain sections of a group of old *Calhm1*^+/+^ (a–c,g–i,m–o) and *Calhm1*^−/−^ (d–f,j–l,p–r) littermates. Hippocampal formation (panels a,d,g,j,m,p), cerebral cortex (b,e,h,k,n,q), and cerebellum (c,f,i,l,o,r) are shown. (**E–H**) Percent area occupied with positive staining for cortical (**E**) CA1 (**F**) and cerebellar (**G**) NeuN expression, and hippocampal (HP) GFAP (**H**) expression, analyzed by immunohistochemistry as in *D* (*n* = 3).

The mouse *Calhm1* gene is located on chromosome 19, extends over 3.1 kb, and contains 2 exons separated by one intron (Fig. 1A). *Calhm1* overlaps at its 3′ end with the putative promoter region of the uncharacterized homolog gene *Calhm2*^1^. We chose the strategy of deleting the *Calhm1* exon 1 to prevent a potential interference with the *Calhm2* promoter. The resulting *Calhm1*^−/−^ mice (Fig. 1B,C) were viable and fertile. They generated litters of normal size, with normal Mendelian inheritance of the mutant allele, ruling out any essential functions of CALHM1 in mouse embryogenesis.

**Figure 2:**
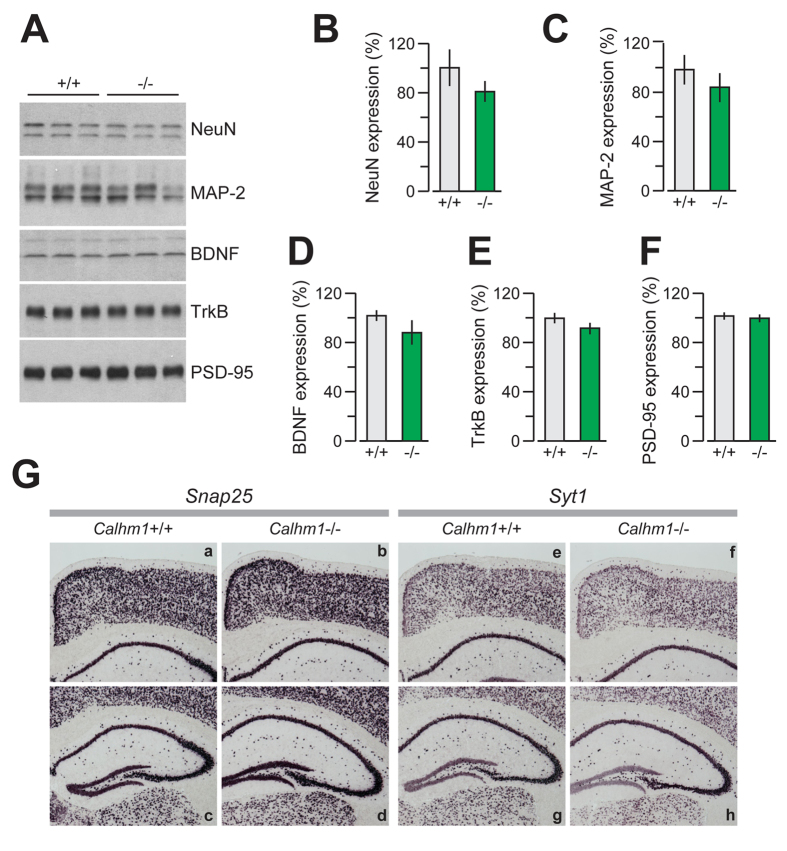
Brain analysis of *Calhm1*^−/−^ mice. (**A**) WB analysis of the levels of the indicated proteins in the whole brain from a group of young-adult *Calhm1*^+/+^ and *Calhm1*^−/−^ littermates. (**B–F**) Densitometric analysis and quantification of the expression levels of the indicated neuronal makers, analyzed by WB as in (**A**) (*n* = 3). (**G**) *In situ* hybridization of *Snap25* (panels a–d) and *Syt1* (e–h) in the cerebral cortex (a,b,e,f) and hippocampus (c,d,g,h) of young-adult *Calhm1*^+/+^ and *Calhm1*^−/−^ mice.

A cohort of adult *Calhm1*^−/−^ and *Calhm1*^+/+^ littermates (see Methods) was sacrificed to examine their brain morphology. Immunohistological analyses of brain sections, using Nissl staining and the neuronal marker protein NeuN, demonstrated that *Calhm1*^−/−^ brains had normal cerebral architecture when compared to WT controls, and displayed no significant alterations of the neuronal populations of the cortex, hippocampus, and cerebellum (Fig. 1D, panels a-l, and Fig. 1E–G). Also, *Calhm1*^−/−^ mice displayed normal immunoreactivity for the glial marker, glial fibrillary acidic protein (GFAP), in these brain regions (Fig. 1D, panels m–r, and Fig. 1H). WB analysis of whole brain homogenates showed no differences between *Calhm1*^−/−^ and *Calhm1*^+/+^ mice in the expression of several neuronal markers, such as NeuN, microtubule-associated protein 2 (MAP-2), brain-derived neurotrophic factor (BDNF), tyrosine receptor kinase B (TrkB), and the synaptic marker post-synaptic density protein 95 (PSD-95; Fig. 2A–F). Furthermore, *in situ* hybridization revealed no alteration in the levels and regional expression of mRNA for the pre-synaptic makers synaptosome-associated protein of 25 kDa (*Snap25*) and synaptotagmin-1 (*Syt1*) in the *Calhm1*^−/−^ hippocampus and cortical brain (Fig. 2G). Thus, *Calhm1*^−/−^ mice presented grossly normal brain cytoarchitecture and showed no evidence of neuronal or glial degeneration, indicating that CALHM1 deficiency did not affect brain development or brain maintenance in adulthood.

### Impaired memory flexibility in *Calhm1* ^−/−^ mice

**Figure 3:**
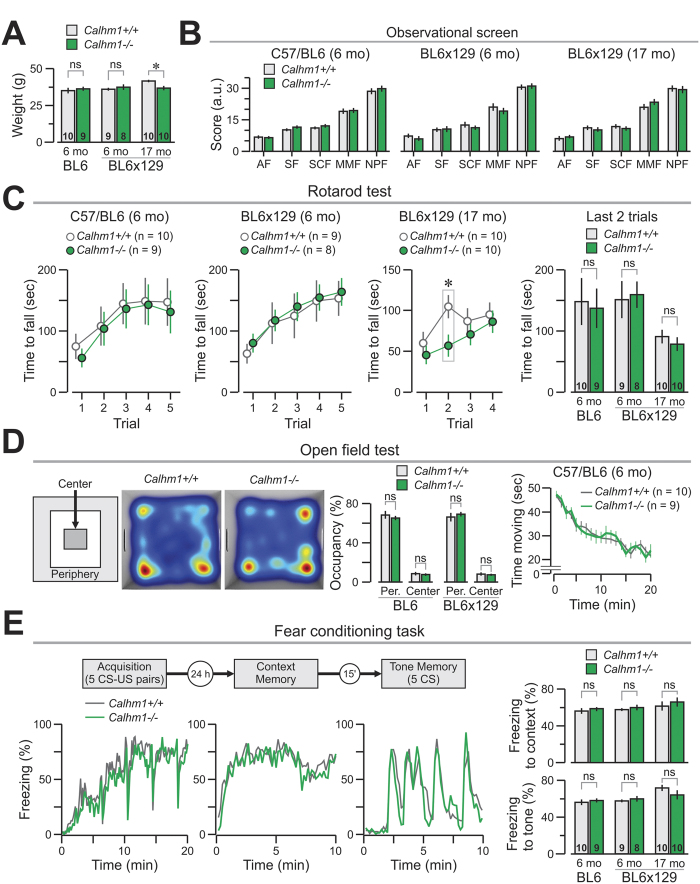
Behavioral assessment of *Calhm1*^−/−^ mice. (**A**) Young-adult animals of both genotypes have similar weights, but old *Calhm1*^−/−^ mice show lower weight than controls, ^*^P < 0.05 (*t* = 2.89, t test). (**B**) Both genotypes behave similarly in the observational screen as shown by their scores for the five functions. (**C**) The rotarod test reveals no differences between genotypes in the time to fall from the rotating drum across the last 2 trials (graph at right), although old *Calhm1*^−/−^ mice show slow motor learning (trial 2 for BL6 × 129 17-mo mice, ^*^P < 0.05, *t* = 2.56, t test). (**D**) Open field test is similar between genotypes. *Left*, top view of the chamber showing the center and periphery (Per.) zones, as well as heat-maps for representative *Calhm1*^+/+^ and *Calhm1*^−/−^ mice during the test. *Middle*, no difference in zone occupancy between genotypes. *Right*, similar time moving during the 20-min test for both genotypes. (**E**) Fear conditioning is equivalent in both genotypes. *Left top*, schematic of the task. *Left bottom*, time courses for the freezing response (% of total time, in 10-sec bins, presented as continuous lines) during the acquisition phase, and the two types of memory testing (context and tone). *Right top*, both genotypes show similar freezing during the last 5 min of the context memory test. *Right bottom*, both genotypes freeze equally during the presentation of the tones in the tone memory test. ns, non significant.

To study whether the deletion of the *Calhm1* gene produced phenotypic alterations, *Calhm1*^−/−^ mice (*n* = 27) and *Calhm1*^+/+^ (*n* = 29) controls were assessed behaviorally at a young-adult and old age (6-mo-old and 17-mo-old, respectively). The animals belonged to either the C57BL/6J strain (6-mo-old, *Calhm1*^−/−^, *n* = 9, *Calhm1*^+/+^, *n* = 10) or the original hybrid 129 × C57 strain (6-mo-old, *Calhm1*^−/−^, *n* = 8, *Calhm1*^+/+^, *n* = 9; 17-mo-old, *Calhm1*^−/−^, *n* = 10, *Calhm1*^+/+^, *n* = 10). Both genotypes performed similarly in an observational screen (Fig. 3B), the rotarod test (Fig. 3C, but notice that old *Calhm1*^−/−^ mice acquired the task more slowly), the open field test (Fig. 3D), and fear conditioning (Fig. 3E), indicating that *Calhm1*^−/−^ mice did not have generalized phenotypic abnormalities. These are important controls because CALHM1 is expressed in the peripheral taste system where it mediates taste perception^16^. Therefore, CALHM1 could have been involved in another sensory mechanism required for basic behavioral responses during cognitive testing.

**Figure 4:**
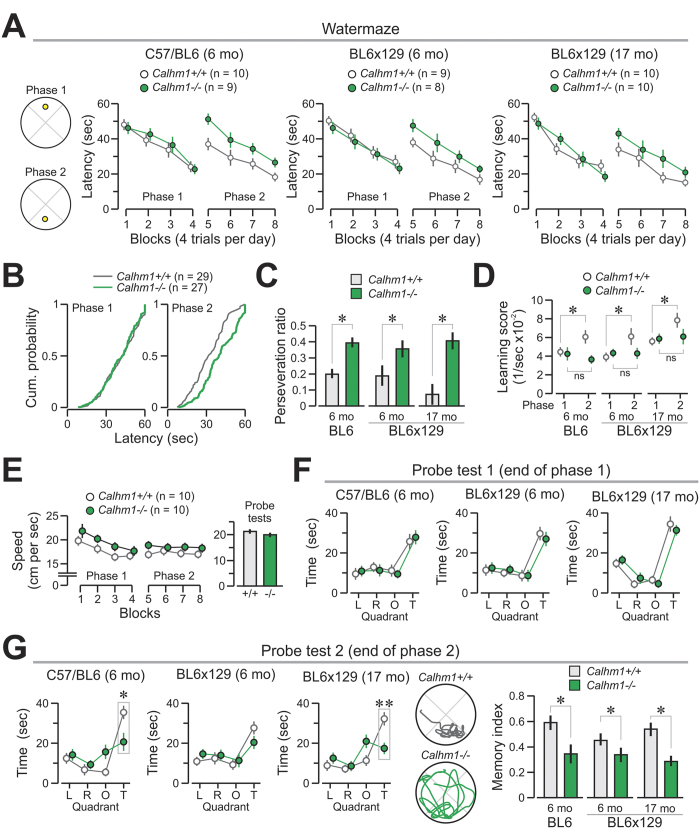
Impaired memory flexibility in *Calhm1*^−/−^ mice. The Morris water maze task was used to assess spatial cognition. **(A)** *Left*, the diagrams show phase 1 of training with the platform (yellow circle) in the North location, and phase 2 with the platform in the South location. *Right*, mice of both genotypes show comparable latencies in phase 1, but *Calhm1*^−/−^ mice display a clear deficit in phase 2, when the platform is switched to a novel location (*left graph*, *F* = 11.9, *P* < 0.001 ; *middle graph*, *F* = 17.8, *P* < 0.001; *right graph*, *F* = 9.54, *P* < 0.005, RMANOVA with last 16 trials as the repeated measure). (**B**) Cumulative probability plots for all trials in each phase show that *Calhm1*^−/−^ mice have significantly longer latencies in phase 2 (*Z* = 2.15, *P* < 0.001, Kolmogorov-Smirnov test). (**C**) The perseveration ratio is markedly higher in *Calhm1*^−/−^ mice. (**D**) Learning scores are unchanged across phases for *Calhm1*^−/−^ mice, whereas *Calhm1*^+/+^ mice show a significantly enhanced score in phase 2. These results show lack of memory flexibility in *Calhm1*^−/−^ mice. (**E**) Swimming speeds are comparable between *Calhm1*^−/−^ and *Calhm1*^+/+^ mice across the two phases (*left*) and probe tests (*right*). (**F**) Performance during the first probe test is similar in both genotypes; abbreviations for pool quadrants, L, left, R, right, O, opposite, T, target. (**G**) Impaired performance of *Calhm1*^−/−^ mice during the second probe test. *Left*, the graphs show lower exploration of the target quadrant by *Calhm1*^−/−^ mice. *Middle*, representative swim-paths of old mice showing focused search by *Calhm1*^+/+^ mouse and broad search by *Calhm1*^−/−^ mouse. *Right*, the spatial memory index is markedly lower in *Calhm1*^−/−^ animals. ^*^P < 0.05; ^**^P < 0.005 (t test).

Notably, both the young-adult and old *Calhm1*^−/−^ groups were significantly impaired in a Morris water maze task for memory flexibility^17^ that required animals to find a hidden platform that was first located in the North quadrant (phase 1) and then in the South quadrant (phase 2) (Fig. 4A). Probe tests were performed at the end of each phase. Mice of both genotypes performed equally on phase 1, in terms of latency (Fig. 4A,B), and swimming speed (Fig. 4E), but showed clear differences in latency during phase 2, whether the mice were in the young-adult or old age group (Fig. 4B, *Z* = 2.15, *P* < 0.001, Kolmogorov-Smirnov test). An analysis centered on the change in location of the platform (from North to South quadrants) revealed that *Calhm1*^−/−^ mice had a significantly higher perseveration ratio (Fig. 4C; *Calhm1*^+/+^, *n* = 29, 0.15 ± 0.03; *Calhm1*^−/−^, *n* = 27, 0.39 ± 0.03; *t* = 5.38, *P* < 0.001, t test) and poorer learning score difference (Fig. 4D; *Calhm1*^+/+^, *n* = 29, 0.0217 ± 0.0035; *Calhm1*^−/−^, *n* = 27, −0.0028 ± 0.0035; *t* = 4.92, *P* < 0.001, t test), revealing a clear deficit in memory flexibility. Additionally, a probe test at the end of phase 1 revealed no differences between genotypes (Fig. 4F), whereas a second probe test at the end of phase 2 (Fig. 4G) showed that, compared to controls, *Calhm1*^−/−^ mice had a reduced spatial bias for their trained quadrant, which was evidenced by their significantly lowered spatial memory index (Fig. 4G; *Calhm1*^+/+^, *n* = 29, 0.53 ± 0.03; *Calhm1*^−/−^, *n* = 27, 0.32 ± 0.03; *t* = 4.67, *P* < 0.001, t test).

### Disrupted LTP in *Calhm1* ^−/−^ mice

**Figure 5:**
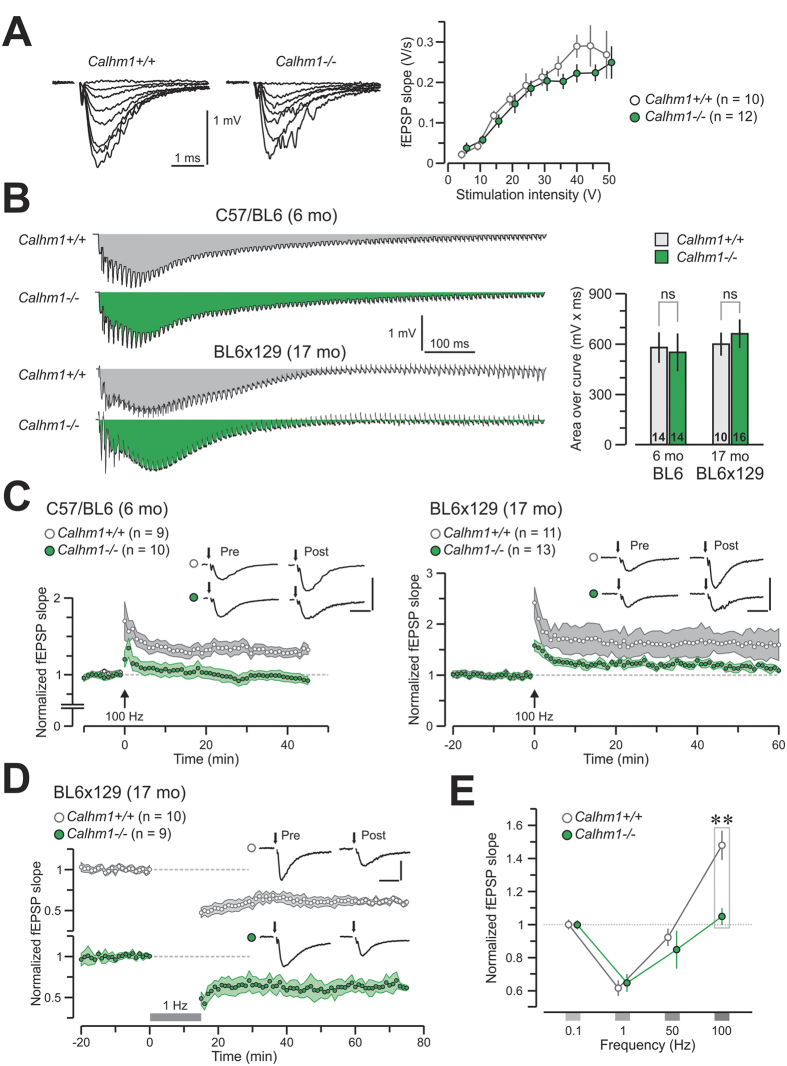
Selective deficit in LTP in *Calhm1*^−/−^ hippocampus. (**A**) *Left*, traces from old *Calhm1*^+/+^ and *Calhm1*^−/−^ mice show representative fEPSPs at increasing stimulation strengths. *Right*, plot displays the mean fEPSP slopes vs. stimulation intensities, revealing comparable input-output functions between genotypes. (**B**) *Left*, representative traces for a train of high-frequency stimulation (HFS, 100 Hz for 1 sec) from young-adult and old *Calhm1*^+/+^ and *Calhm1*^−/−^ mice, in which the stimulus artifacts have been subtracted. Shaded areas-over-the-curves are used for analysis. *Right*, total integral of HFS train is comparable across groups. (**C**) LTP is impaired in young-adult and old *Calhm1*^−/−^ mice. A comparison at 45 min post-HFS reveals significant differences between genotypes (*left*, *t* = 4.23, *P* < 0.0001; *right*, *t* = 3.42, *P* < 0.005, t test). *Inset*, traces at 5 min pre- and 45 min post-HFS. (**D**) LTD is not affected in old *Calhm1*^−/−^ mice. *Inset*, traces at 5 min pre- and 60 min post-LFS, low-frequency stimulation (1 Hz for 15 min). (**E**) BCM curves showing selective deficit of LTP expression (100 Hz train) in *Calhm1*^−/−^ mice. Each point represents the mean ± SEM at 60 min post-1 Hz, 30 min post-50 Hz, and 45 min post-100 Hz; range = 9–23, young-adult and old experiments combined for each genotype. ^*^P < 0.01, t test. For (**C,D**), arrows indicate blanked stimulus artifacts; scale, x-axis, 10 msec, y-axis, 1 mV.

To examine whether synaptic function was altered in young-adult and old *Calhm1*^−/−^ mice, electrophysiological studies were conducted^17^,^18^ in *ex vivo* slices from the hippocampus, a brain region that is critically involved in memory encoding. Basal synaptic function was examined by recording field excitatory post-synaptic potentials (fEPSP) between CA3–CA1 synapses. Input-output (I-O) functions with stimulation intensity of the CA3 axons as the input and fEPSP slope as the output were similar between genotypes (Fig. 5A; *Calhm1*^+/+^, *n* = 10; *Calhm1*^−/−^, *n* = 12; *F* = 0.19, *P* = 0.67, RMANOVA). Analysis of synaptic burst responses produced by high-frequency stimulation (HFS) of CA3 axons showed that the integral of the burst responses was similar between genotypes (Fig. 5B; *Calhm1*^+/+^, *n* = 24; *Calhm1*^−/−^, *n* = 30; *t* = 0.24, *P* = 0.8, t test). These results demonstrate that the absence of CALHM1 does not affect the basal function of excitatory CA1 synapses.

Synaptic plasticity was assessed with LTP and long-term depression (LTD) studies in *ex vivo* hippocampal slices^19^, prepared from *Calhm1*^−/−^ mice and *Calhm1*^+/+^ controls. Remarkably, *Calhm1*^−/−^ slices exhibited a significant deficit in LTP when compared to *Calhm1*^+/+^ slices, measured 45 min after HFS delivery (Fig. 5C; *Calhm1*^+/+^, *n* = 20, 147.97 ± 8.76%; *Calhm1*^−/−^, *n* = 23, 104.98 ± 3.96%; *t* = 4.47, *P* < 0.0001, t test). Moreover, measurement of short-term potentiation (20 min after HFS) indicated that *Calhm1*^−/−^ slices were also deficient at this earlier point (*Calhm1*^+/+^, *n* = 20, 166.87 ± 5.2%; *Calhm1*^−/−^, *n* = 23, 116.53 ± 5.2%; *t* = 4.13, *P* < 0.0001, t test). In contrast, *Calhm1*^−/−^ slices exhibited normal LTD when compared to *Calhm1*^+/+^ slices, measured 60 min after LTD induction (Fig. 5D; *Calhm1*^+/+^, *n* = 10, 63.9 ± 0.4%; *Calhm1*^−/−^, *n* = 9, 64.4 ± 0.7%; *t* = 0.55, *P* = 0.59, t test). In addition, we used the induction trains for LTP (100 Hz for 1 sec) and LTD (1 Hz for 15 min), together with an intermediate train (50 Hz for 2 sec) to construct a “BCM curve”^19^ (Fig. 5E), which clearly showed a shift to the right in the frequency dependence of synaptic plasticity in the *Calhm1*^−/−^ group and highlighted the selective deficit in LTP expression for *Calhm1*^−/−^ mice.

Notably, we studied the *Calhm1*^−/−^ mice in the original hybrid and C57BL/6J backcrossed genetic backgrounds (see Methods), as well as at the young-adult and old stages. Our behavioral and electrophysiological studies revealed that the same deficits due to CALHM1 deficiency were present in the Morris water maze task and LTP measurements (Figs 3, 4, 5). In combination, these data show that CALHM1 controls memory flexibility and plays a selective role in the molecular cascade responsible for the plastic enhancement of CA1 synapses but not in the molecular cascade linked to LTD.

### CALHM1 activation controls PKA-mediated NMDAR and AMPAR phosphorylation and glutamate-mediated c-Fos and C/EBPβ expression in neurons

**Figure 6:**
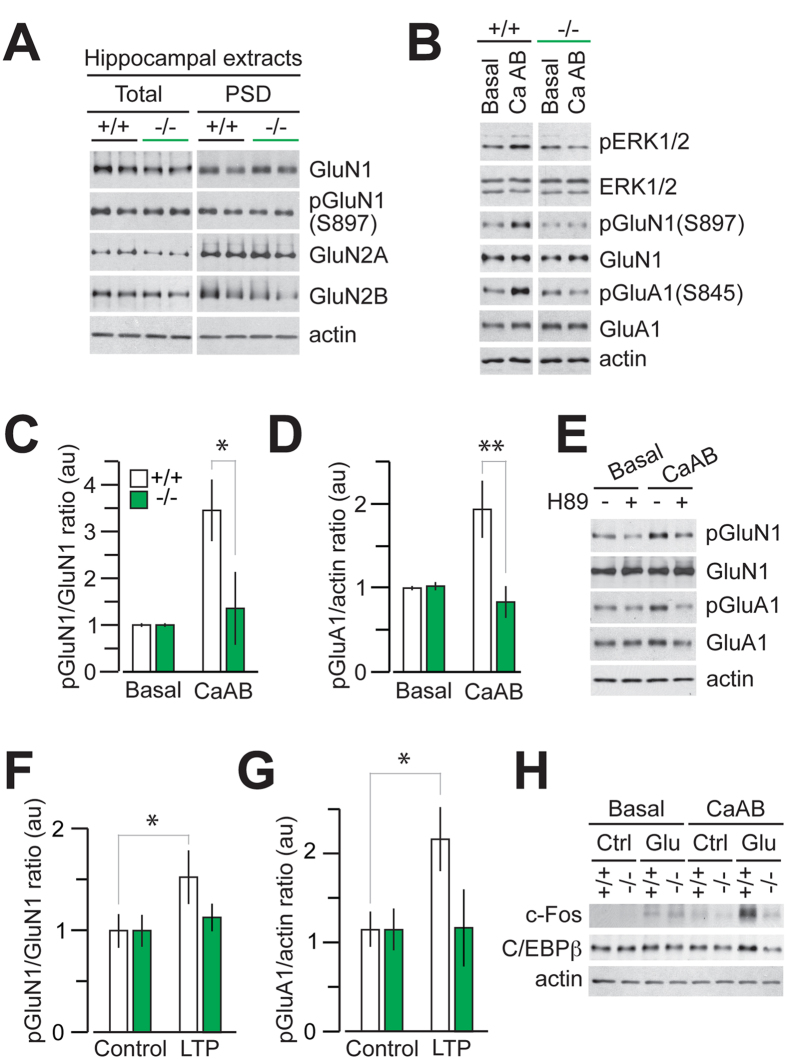
CALHM1 activation controls PKA-mediated NMDAR and AMPAR phosphorylation and glutamate-mediated c-Fos and C/EBPβ expression in neurons. (**A**) WB analysis of the levels of the indicated proteins in whole hippocampal homogenates (Total) and PSD fractions obtained from *Calhm1*^+/+^ and *Calhm1*^−/−^ mice. (**B**) Primary neurons isolated from *Calhm1*^+/+^ and *Calhm1*^−/−^ mice were challenged with the calcium add-back condition (CaAB) or not (Basal). Cell extracts were analyzed by WB for the indicated proteins. Representative results from 4 independent experiments are depicted. (**C,D**) Densitometric analysis and quantification of the ratio for phospho-Ser-897 GluN1 over total GluN1 (pGluN1/GluN1), (**C**) and for phospho-Ser-845 GluA1 over actin (pGluA1/actin), (**D**) from *Calhm1*^+/+^ and *Calhm1*^−/−^ primary neurons treated as in (**B**). au, arbitrary units (*n* = 6; ^*^P < 0.05; ^**^P < 0.01; t test). (**E**) WB analysis of the levels of the indicated proteins in *Calhm1*^+/+^ primary neurons pretreated for 30 min with H89 (10 μM) and then challenged with CaAB, as in (**B**). (**F,G**) Ratio for phospho-Ser-897 GluN1 over total GluN1 (pGluN1/GluN1), (**F**) and for phospho-Ser-845 GluA1 over actin (pGluA1/actin), (**G**) from LTP-stimulated hippocampal slices (*n* = 10–12; ^*^P < 0.05; t test). (**H**) WB analysis of the levels of the indicated proteins in *Calhm1*^+/+^ and *Calhm1*^−/−^ primary neurons challenged with CaAB, as in (**B**), in the absence (Ctrl) or presence of glutamate stimulation (Glu, 20 μM, 1 h incubation for c-Fos, 4 h for c/EBPβ).

The observed impairment of LTP by CALHM1 deficiency suggests that CALHM1 cross talks with signaling mechanisms relevant to synaptic activity. WB analyses of whole hippocampal homogenates and postsynaptic density (PSD) fractions revealed no changes in the levels of the N-methyl-D-aspartate receptor (NMDAR) subunits, GluN1, GluN2A, and GluN2B, in *Calhm1*^−/−^ mice (Fig. 6A), showing that CALHM1 deficiency did not affect NMDAR expression or stability. Our previous work has showed that CALHM1 activation in primary neurons (using the CaAB condition) can stimulate intracellular calcium signaling^12^. One consequence of this stimulation is the increase of the ERK1/2 signaling cascade in neurons [Fig. 6B and ref. 12]. Intracellular calcium elevations can lead to the activation of multiple other signaling kinases^20^,^21^, including protein kinase A (PKA), which plays a critical role in activity-dependent gene expression in neurons^22^. For instance, PKA controls NMDAR trafficking by phosphorylating Ser-897 on GluN1^23^,^24^. Importantly, we found that CaAB resulted in a significant elevation of phospho-Ser-897 GluN1 levels in *Calhm1*^+/+^ primary neurons but not in *Calhm1*^−/−^ neurons (Fig. 6B,C). Pretreatment with the PKA inhibitor H89 prevented the effect of CaAB on phospho-Ser-897 GluN1 (Fig. 6E). Another well-established target of PKA during synaptic activity is the α-amino-3-hydroxy-5-methyl-4-isoxazolepropionate receptor (AMPAR), which is phosphorylated at Ser-845 on the GluA1 subunit^25^,^26^. Similarly to NMDAR phosphorylation, AMPAR phosphorylation at Ser-845 on GluA1 was significantly increased by CaAB in *Calhm1*^+/+^ neurons. This effect on phospho-Ser-845 GluA1 was absent in *Calhm1*^−/−^ neurons (Fig. 6B,D) and was fully prevented by H89 (Fig. 6E). Altogether these data show that CALHM1 activation controls NMDAR and AMPAR phosphorylation by PKA.

To determine whether CALHM1 controls NMDAR and AMPAR phosphorylation in a more physiologically relevant system, we asked whether CALHM1 deficiency affects the levels of phospho-Ser-897 GluN1 and phospho-Ser-845 GluA1 in LTP-stimulated hippocampal slices. In whole hippocampal homogenates at steady states, we could not observe any changes in phospho-Ser-897 GluN1 levels in *Calhm1*^−/−^ mice (Fig. 6A). However, we found that following LTP, phospho-Ser-897 GluN1 and phospho-Ser-845 GluA1 levels in *Calhm1*^+/+^ hippocampal slices were significantly increased (compared to non-tetanized slices), whereas LTP had no effect on GluN1 and GluA1 phosphorylation in slices obtained from *Calhm1*^−/−^ mice (Fig. 6F,G). These results demonstrate that CALHM1 controls NMDAR and AMPAR phosphorylation in brain slices in response to a physiologically relevant stimulus that activates PKA and synaptic activity.

To go further, we then asked whether CALHM1 is required for glutamate-mediated immediate-early gene (IEG) expression in neurons. We found that CaAB-triggered CALHM1 activation in *Calhm1*^+/+^ primary neurons robustly potentiated the effect of glutamate on the expression of c-Fos and also—but to a lesser extent—of C/EBPβ (Fig. 6H), two IEG markers of neuronal activity^22^. Strikingly, this effect was absent in *Calhm1*^−/−^ neurons (Fig. 6H), demonstrating that CALHM1 is required for the control of c-Fos and C/EBPβ expression by glutamate exposure in neurons. Thus, CALHM1 activation controls PKA-mediated NMDAR and AMPAR phosphorylation and glutamate-mediated IEG expression in neurons.

## Discussion

In this study, we provide the first characterization of CALHM1 function in synaptic activity and cognition in mice. Using behavioral and electrophysiological studies, we reveal that *Calhm1*^−/−^ mice display significant deficits in LTP and memory flexibility. We further show that under conditions of synaptic activation, CALHM1 controls the phosphorylation by PKA of two key mediators of synaptic transmission in cerebral neurons, NMDAR and AMPAR. Moreover, we demonstrate that CALHM1 potentiates the effect of glutamate on c-Fos and C/EBPβ expression, two markers of neuronal activity. Altogether, these data identify CALHM1 as a novel regulator of neuronal signaling during synaptic activity, which is required for the proper expression of memory flexibility.

The molecular mechanisms of memory flexibility are incompletely understood. Here we show that CALHM1 is required for both memory flexibility and PKA-mediated NMDAR and AMPAR phosphorylation in cerebral neurons. Phosphorylation of NMDAR and AMPAR by PKA is a critical regulatory mechanism of these receptors during activity-dependent synaptic function. NMDAR is essential for the induction of synaptic plasticity and memory formation^27^, and NMDAR subunit phosphorylation controls the trafficking of this receptor and ultimately its function^28^. Recently, we have reported that CALHM1 channel activation in neuronal cells primarily signals through the MEK/ERK/MSK/RSK kinase signaling cascade^12^. A cross talk between ERK1/2 and PKA is also observed during CALHM1 activation^12^, suggesting that CALHM1 controls neuronal PKA. PKA targets NMDAR by phosphorylating GluN1 at Ser-897^28^. Recent evidence, obtained in S897A-GluN1 knock-in mice, demonstrates that this phosphorylation is required for both NMDAR- and AMPAR-mediated synaptic transmission and LTP^29^. These results are in line with the deficits observed in *Calhm1*^−/−^ mice and suggest that the reduced levels in GluN1 phosphorylation at Ser-897 might be causally linked to the impaired LTP caused by CALHM1 deficiency. Our work therefore sheds light on a new mechanism for controlling memory flexibility.

During synaptic activity, PKA also phosphorylates the GluA1 subunit of the AMPAR at Ser-845 to influence channel function and trafficking^30^,^31^,^32^,^33^. GluA1 phosphorylation at Ser-845 has been previously linked to LTD and fear memory^34^,^35^. In our study, CALHM1 deficiency decreases GluA1 phosphorylation at Ser-845 during synaptic activation; however, *Calhm1*^−/−^ slices do not show any deficit in LTD. This decoupling suggests that the effect of CALHM1 deficiency on AMPAR phosphorylation is not a central event in its effect on memory. Further studies are required to delineate the exact cross talk between NMDAR and AMPAR phosphorylation during CALHM1-dependent memory formation.

A recent study has already reported results on the effect of CALHM1 deficiency on spatial memory and learning in another *Calhm1*^−/−^ mouse model^11^. Cognition in these mice was assessed by using a version of the Morris water maze, which only included the phase 1 of the corresponding task reported in this manuscript (see Fig. 4A). In line with our results, the authors found that CALHM1 deficiency did not affect cognition in phase 1 of the Morris water maze, see Fig. 4A and ref. 11. Memory flexibility was not investigated in this study^11^.

The specific effect of CALHM1 deficiency on memory malleability is of interest. Indeed, memory flexibility relates to episodic memory and its deterioration is an early and invariable manifestation of AD^36^,^37^. The molecular trigger for episodic amnesia at the early stages of AD is unknown. However, several studies have identified defects in brain activity in specific regions, such as the medial temporal and frontal lobes^38^,^39^. It will be interesting to determine whether such defects in memory encoding are also apparent in *Calhm1*^−/−^ mice. This would suggest that CALHM1 loss-of-function in these brain regions might contribute to episodic amnesia at the early stages of AD pathogenesis.

How CALHM1 at the molecular level might influence AD onset is unclear. We have previously reported that CALHM1 expression in cell lines represses the accumulation of Aβ^1^,^5^,^6^. Two independent genetic studies have showed that a CALHM1 variant (P86L) influences Aβ levels in human cerebrospinal fluid^8^,^9^. *In vitro* functional studies further demonstrated that the CALHM1 P86L variant caused a partial loss of CALHM1 function by interfering with CALHM1 ion channel properties and by de-repressing its effect on Aβ accumulation^1^,^12^,^13^,^40^,^41^. Collectively, these results support the notion that CALHM1 might control both Aβ metabolism and AD pathogenesis. In the context of the present work, future studies will have to determine whether the observed cognitive deficits in *Calhm1*^−/−^ mice are mediated, at least in part, by a deregulation in Aβ homeostasis. For example, it will be important to investigate whether CALHM1 deficiency leads to an increase in Aβ levels in brain regions affected by episodic amnesia, such as the medial temporal or frontal lobes. At the molecular level, it will also be interesting to determine whether Aβ cross talks with CALHM1 signaling to control PKA-mediated NMDAR and AMPAR phosphorylation and function during synaptic activity and memory flexibility formation.

## Materials and Methods

### *Calhm1* knockout (KO) mice

All animal experiments were performed according to procedures approved by the Feinstein Institute for Medical Research and Monell Chemical Senses Center Institutional Animal Care and Use Committees. *Calhm1*^+/−^ breeders were generated at genOway. Deletion was performed by homologous recombination in cells using the PMA1-HR targeting vector (genOway). The targeting vector was electroporated into 129Sv embryonic stem (ES) cells and 307 resistant ES cell clones were isolated, screened by PCR, and confirmed by Southern blot analysis to unambiguously confirm the 5′ and 3′ targeting events. Selected ES cell clones were injected into C57BL/6J blastocytes that were then re-implanted into OF1 pseudo-pregnant females and allowed to develop to term. Successful germline transmission was achieved and agouti pups containing the recombined allele were obtained. Wild type (WT, *Calhm1*^+/+^) and *Calhm1* KO (*Calhm1*^−/−^) littermates of the F2 generation (hybrid 129Sv × C57BL/6J genetic background) were used in this study. Some *Calhm1*^−/−^ hybrid mice were backcrossed with C57BL/6J mice bearing the *EIIa–cre* transgene (B6.FVB-Tg (EIIa-cre)C5379Lmgd/J, Jackson Lab) to remove the neomycin resistance cassette by *cre*-loxP-mediated excision (see schematic representation in Fig. 1A). The resulting *Calhm1*^+/−^ mice were then backcrossed for 10 generations into the C57BL/6J background, before being made homozygous. The removal of the neomycin cassette was confirmed by PCR. Two age groups of mice carrying the original hybrid genetic background, young-adult (3–6-mo-old) and old (15–17-mo-old), and one group of young-adult backcrossed mice (6-mo-old), were analyzed in this study.

### Chemicals and antibodies

H89 and antibodies directed against NeuN and C/EBPβ were purchased from EMD Millipore. Antibodies directed against ERK1/2, phospho-ERK1/2 (pERK1/2, Thr-202/Tyr-204), pGluN1 (Ser-897), GluN2A, GluN2B, pGluA1 (Ser-845), and c-Fos were from Cell Signaling Technology. Anti-actin antibody was from BD Transduction Laboratories. The anti-GluN1 (clone N308/48) and anti-GluA1 (N355/1) monoclonal antibodies were obtained from the UC Davis/NIH NeuroMab Facility.

### Brain histochemistry

Sagittal sections (5 μm thick) of formalin-fixed paraffin-embedded brain tissue samples were Nissl-stained with cresyl violet or immunostained with anti-NeuN (1:100 dilution) and anti-GFAP (1:100) antibodies. Immunohistochemistry was performed as described before^42^, with the following modifications. Sagittal sections of formalin-fixed paraffin-embedded brain tissue were deparaffinized by immersion in xylene and hydration through graded ethanol solutions. Endogenous peroxidase activity was inhibited by incubation in 5% hydrogen peroxide in TBS-T for 30 min at room temperature (RT). After washing twice in TBS-T for 5 min, sections were blocked in 5% fat-free milk in TBS-T for 1 h at RT. Sections were then incubated in the presence of primary antibodies diluted in 5% fat-free milk in TBS-T overnight at 4 °C in a humidified chamber. After washing, the sections were incubated with biotin-coupled anti-mouse IgG1 secondary antibodies (1:1,000 dilution in TBS-T with 20% Superblock, Thermo Fisher Scientific) before incubation with streptavidin-horseradish peroxidase (1:1,000 dilution in 20% Superblock TBS-T, Southern Biotech) and visualization with diaminobenzidine tetrahydrochloride. For *in situ* hybridization, we used methods described previously^16^,^43^. In brief, 10-μm-thick coronal sections of fresh-frozen brains were fixed with 4% PFA, treated with diethylpyrocarbonate, and hybridized with antisense riboprobe at 58 °C. After hybridization, the sections were washed in 0.2 × SSC at 58 °C and incubated with alkaline phosphatase-conjugated anti-digoxigenin antibody (1:500, Roche Diagnostics). Signals were visualized with 4-nitro blue tetrazolium chloride/5-bromo-4-chloro-3-indolyl-phosphate for 16 h at RT. RNA probes generated were to nucleotides 96–1734 of *Snap25* (GenBank accession number BC018249) and 1–3789 of *Syt1* (GenBank accession number BC042519).

### PSD isolation and Western blot (WB) analyses

Forebrains of *Calhm1*^+/+^ or *Calhm1*^−/−^ mice were homogenized in homogenization buffer (10 mM HEPES, pH 7.4, 320 mM sucrose) containing proteases and phosphatases inhibitors (Roche Applied Science). Cells debris and nuclei were removed by 1,000 × g centrifugation. The supernatant was spun for 20 min at 12,000 × g, resulting in supernatant and P2 pellet. The latter was resuspended in a buffer containing 4 mM HEPES, 1 mM EDTA, proteases and phosphatases inhibitors, and spun for 20 min at 12,000 × g. The resulting pellet was resuspended again in HEPES buffer and spun for 20 min at 12,000 × g. The resulting pellet was resuspended in a buffer containing 20 mM HEPES, pH 7.2, 100 mM NaCl, 0.5% Triton X-100, proteases and phosphatases inhibitors, and rotated slowly for 15 min before being spun for 20 min at 12,000 × g. The supernatant was used as the non PSD fraction and the pellet was resuspended in a buffer containing 20 mM HEPES, pH 7.5, 0.15 mM NaCl, 1% Triton X-100, 1% deoxycholic acid, 1% SDS, 1 mM DTT, proteases and phosphatases inhibitors, allowed to rotate gently for 1 h and spun for 15 min at 10,000 × g. The resulting supernatant was used as the PSD fraction. For WB analyses, whole brain and PSD extracts were separated by SDS-PAGE and transferred to nitrocellulose membranes, as described before^12^.

### *Ex vivo* electrophysiology

We have published these methods elsewhere^17^,^18^. Briefly, mice were anesthetized with isoflurane in a mobile anesthesia chamber, then immediately decapitated. The brain was quickly extracted and placed in ice-cold (<2 °C) artificial cerebral spinal fluid (ACSF) containing the following (mM): 126 NaCl, 26 NaHCO_3_, 10 glucose, 2.5 KCl, 2.4 CaCl_2_, 1.3 MgCl_2_, 1.2 NaH_2_PO_4_, and 1 kynurenic acid, constantly gassed with 95% O_2_, 5% CO_2_ (“carbogen”). The brain was bisected then mounted on a block with ethyl cyanoacrylate glue. Transverse hippocampal slices (400 μm) were cut on a Leica VT1200 brain slicer while bathed in ice-cold ACSF. Slices were incubated (35 °C for 35 min), then allowed to equilibrate back to 25 °C for at least 2 h. For *ex vivo* field recordings, slices were transferred into a recording chamber continuously perfused with 30 °C ACSF. Picrotoxin (100 μM) was added to block GABA_A_-mediated activity. Field excitatory postsynaptic potentials (fEPSPs) were recorded with borosilicate glass electrodes (2–3 MΩ tip resistance) placed in hippocampal area CA1’s *stratum radiatum*. Two bipolar Pt-Ir stimulating electrodes (Frederick Haer & Co., Bowdoinham, ME) were placed over the Schaeffer collateral/commissural axons, so that we could activate (Grass SD9 square-voltage-pulse stimulator) two independent pathways, test and control, in the same slice. To obtain baseline responses, each pathway was stimulated every 10 sec (0.1 Hz). The fEPSPs were amplified (AM Systems 1800), digitized, and stored on a PC running acquisition software (custom programs based on AxoBasic, or WinLTP v2, Bristol, UK). For the input-output functions, stimulation intensity was reduced to a value at which no fEPSP was evoked, and the stimulation was then increased incrementally to elicit fEPSPs until a population spike was detected, which defined the final point of the function. For burst analysis, a stable baseline was recorded for ~10 min and then a single HFS train (100 Hz for 1 sec) was delivered and analyzed offline (Origin v9, OriginLab) by subtracting the stimulus artifacts and integrating the total area-over-the-curve of the response. For 50 Hz trains, following a baseline period (~10 min) a single train (50 Hz for 2 sec) was delivered and responses were further recorded for at least 30 min. For LTP experiments, a stable baseline was recorded (~15 min), followed by one HFS train to induce LTP and at least 45 min of post-HFS responses. For LTD experiments, the baseline recording (~15 min) was followed by LTD induction (LFS protocol of 1 Hz for 15 min, 900 pulses total) and at least 70 min of post-LFS responses.

### Morris water maze task

We have published these methods previously^17^. Importantly, animals were maintained in a reverse lighting schedule (lights on at 9:00AM, lights off at 9:00PM) and were assessed in all the behavioral procedures during the dark cycle. We used a water maze (diameter, 160 cm), which was filled with water (18–20 °C) and surrounded by focally illuminated distal cues that were mounted on the room walls. We used behavioral software (Ethovision v8.5, Noldus) to track and record the animal movement. Each mouse was trained to find a hidden escape platform (diameter, 10 cm) that was submerged 0.5 cm below the surface of the water. A trial was terminated when the animal located the platform, or 60 sec elapsed, in which case the mouse was guided to the platform and allowed to sit on it for ~10 sec. For phase 1, mice received 4 trials per day for 4 days with the platform located at the center of the North quadrant. For phase 2, there were 4 trials per day for 4 days with the platform at the center of the South quadrant.

For analysis, the latencies to find the platform were grouped in blocks of 4 trials each. The perseveration ratio was calculated as the latency of the last 4 trials in phase 1 (L1) subtracted from the latency of the initial 4 trials in phase 2 (L2), divided by their sum [(L2 − L1)/(L1 + L2)]. The learning score was computed as the average of the inverse-of-latency during non-novel blocks (i.e., for phase 1, blocks 2 to 4, for phase 2, blocks 6 to 8). After each phase, mice received a probe trial (duration, 60 sec) in which the platform was removed entirely. To analyze the probe trials, the pool was divided into four imaginary quadrants and the time spent in each sector was measured. The spatial memory index was defined as the mean fraction of total time in the Target quadrant during the probe trial.

### Behavioral assessments

Besides the Morris water maze task, all mice were subjected to an observational screen, adapted from ref. 44 and the first stage of the SHIRPA procedure^45^, the rotarod task, the open field test, and fear conditioning. The tests were separated by at least 1 day and conducted during the dark cycle.

The observational screen started with anatomical parameters (coat length, hair length and hair morphology), followed by observation in a cylindrical glass flask (height 15 cm, diameter 11 cm), which measured body position, spontaneous activity, respiratory rate, tremor occurrence, defecation and urination. Transfer to an arena (55 cm × 33 cm) allowed for measuring of transfer arousal, latency to move in the arena and locomotion in the arena. This was continued with manipulations for measuring piloerection, palpebral closure, startle response, gait, pelvic elevation, tail elevation, touch escape, positional passivity, trunk curl, limb grasping, visual placing, grip strength, body tone, pinna reflex, corneal reflex, toe pinch, body length, tail length, lacrimation, whisker morphology, provoked biting, salivation, heart rate, abdominal tone, skin color and limb tone. Measuring several reflexes (wire maneuver, righting reflex, contact righting, negative geotaxis) completed the screen. Throughout the screen, incidences of fear to the experimenter, irritability, aggressivity to the experimenter, vocalizations and abnormal behavior were recorded. Finally, body weight was measured. We found that CALHM1 deficiency did not affect body weight in the young-adult mouse groups, but slightly reduced it in the old group (Fig. 3A), as recently reported^46^. The observed parameters were grouped according to five functional categories^45^, which were: muscle and spinal function; spinocerebellar function; sensory function; neuropsychiatric function; and autonomic function. The summed scores for each function were averaged across mice belonging to the same group (*Calhm1*^+/+^ or *Calhm1*^−/−^) and these were then subjected to statistical analysis.

For the rotarod test, mice were placed individually on a rotating drum (ENV-576M, Med Associates Inc, St. George, VT, USA), which accelerated from 4 to 40 rpm over a course of 5 min. The time at which the mouse fell off the drum was recorded. The test was repeated 4 times for each mouse with an interval of at least 1 h between trials. The room was illuminated with low-level white lights.

The open field test^47^ consisted of a single trial (20 min) in which each mouse was placed in a square chamber (40 cm on the side, 30-cm high walls painted gray) with bedding on the floor. The mouse was transported into the darkened experimental room and immediately placed in the experimental chamber. We used behavioral software (Ethovision v8.5) to track the position and movement of the animal. For analysis, a center zone (10-cm square at the center of the chamber) and a periphery zone (7-cm wide corridor adjacent to the walls) were defined (Fig. 3D); the occupancy in these zones was computed and expressed as the percent of total time. The animal’s movement was calculated at 60 sec intervals.

The fear conditioning task^48^ was implemented in a conditioning chamber (clear Plexiglas, dim light, metal grid floor) and a testing chamber (dark plastic, brightly lit, black floor). Video cameras were mounted on top of the chambers for videotaping and control of stimuli by software (FreezeFrame). The procedure was as follows: on the day before conditioning (day 1), mice were habituated to both chambers for 10 min in a counterbalanced manner to control for order effects. On the day of conditioning (day 2), each mouse was acclimated to the conditioning chamber (3 min) and then given five pairings of a conditional stimulus (tone, 20-sec long, 5 kHz, 80 dB) that co-terminated with an unconditional stimulus (foot shock, 1 sec, 1 mA). The inter-trial interval was 90–120 sec. On the day of testing (day 3), the freezing responses to the conditional stimulus were measured in the testing chamber with five test tones (20 sec, 5 kHz, 80 dB, 100-sec interval), starting from the first tone and lasting until 100 sec after the fifth tone. The freezing response was expressed as the percent of the total time that the animal remained frozen. After 1 h, the mice were placed in the conditioning chamber and were allowed to explore for 5 min (to give them time to recognize the context), after which the duration of freezing was scored for an additional 5 min.

### Primary neuronal culture preparation and treatments

Primary neurons were prepared as described previously^49^. 7–9 DIV neurons were used for the different treatments. CaAB was performed as described previously^1^,^12^. Briefly, cells were incubated for 10 min in Ca^2+^/Mg^2+^-free Hank’s balanced salt solution (HBSS), supplemented with 20 mM HEPES buffer, 0.5 mM MgCl_2_, and 0.4 mM MgSO_4_. Calcium was then added back for 10 min to a final concentration of 1.8 mM. Cells were washed after the different treatments, homogenized, and analyzed by WB.

### Semi-quantitative PCR

*Calhm1* expression levels in brain tissue were determined by PCR following procedures described previously^12^.

### Statistical Analysis

Datasets are presented as mean ± SEM. We used factorial ANOVA, repeated measures ANOVA, Kolmogorov-Smirnov test, and the Student t test to examine statistical significance, which was defined as *P* < 0.05.

## Additional Information

**How to cite this article**: Vingtdeux, V. *et al*. CALHM1 deficiency impairs cerebral neuron activity and memory flexibility in mice. *Sci. Rep*. **6**, 24250; doi: 10.1038/srep24250 (2016).
